# Maresin-1 and S-Equol as Emerging Metabolic Biomarkers in Gestational Diabetes-Associated Inflammation

**DOI:** 10.3390/diagnostics15192439

**Published:** 2025-09-25

**Authors:** Seyda Yavuzkir, Derya Kardas Cinar, Ahmet Cinar, Furkan Bildirici, Suleyman Aydin

**Affiliations:** 1 Department of Obstetrics and Gynecology, Medical School, Firat University, Elazig 23119, Turkey; syavuzkir@firat.edu.tr; 2 Department of Obstetrics and Gynecology, Bismil State Hospital, Diyarbakir 21500, Turkey; kardasderya5@gmail.com (D.K.C.); dr.ahmetcinar@gmail.com (A.C.); 3 Firat Hormones Research Group, Department of Medical Biochemistry and Clinical Biochemistry, Medical School, Firat University, Elazig 23119, Turkey; fbildirici@firat.edu.tr

**Keywords:** gestational diabetes, trimethylamine-N-oxide, Maresin-1, S-Equol, C-reactive protein, indoxyl sulfate

## Abstract

**Background/Objectives:** The most prevalent metabolic condition during pregnancy is gestational diabetes mellitus (GDM), typically diagnosed in the second or third trimester and absent prior to gestation, with a reported prevalence ranging between 1% and 14%. Although the pathogenesis of GDM is thought to involve increased insulin resistance, impaired beta-cell function and mass, and a heightened inflammatory state, the underlying pathophysiological mechanisms remain incompletely understood. Thus, the purpose of this study was to look into any possible relationships between GDM and particular inflammatory biomarkers (Maresin-1 [MaR-1], high-sensitivity-C-reactive protein [Hs-CRP]) as well as microbiota-derived metabolites (Trimethylamine-N-oxide [TMAO], S-Equol, and Indoxyl Sulfate [IS]). **Methods:** A total of 44 pregnant women were enrolled in this study, comprising 22 women with GDM and 22 healthy pregnant controls. Venous blood samples were collected, and serum levels of TMAO, IS, Hs-CRP, MaR-1, and S-Equol were quantified using enzyme-linked immunosorbent assay (ELISA). **Results:** Serum levels of MaR-1 and S-Equol were significantly reduced in the GDM group compared to healthy controls (*p* < 0.05). In contrast, no statistically significant differences were observed in the levels of TMAO, IS, or Hs-CRP between the GDM and control groups (*p* > 0.05). **Conclusions:** The observed reductions in MaR-1 and S-Equol levels among GDM patients suggest a potential role for these anti-inflammatory mediators in the inflammatory processes associated with GDM. That is, these findings imply that the advantages of using these MaR-1 and S-Equol could be predictive for GDM.

## 1. Introduction

Pregnancy is a physiological state characterized by hyperglycemia due to the placental secretion of diabetogenic hormones such as growth hormone, prolactin, human placental lactogen, progesterone, and corticotropin-releasing hormone [1]. Gestational diabetes mellitus (GDM) was first described by Jørgen Pedersen in 1952 [2]. This disease is the most prevalent metabolic disorder in pregnancy, affects approximately 5–25% of all pregnancies [3]. In late gestation, peripheral insulin sensitivity decreases by approximately 50–60% [4]. In healthy pregnancies, insulin production increases two- to threefold to maintain normoglycemia [5]. As a result, insulin resistance physiologically increases during pregnancy. To compensate for decreased insulin sensitivity and increased insulin demands, adaptive pancreatic beta-cell function is upregulated [6]. However, in some women—either previously normoglycemic or with impaired glucose tolerance—this compensatory mechanism may become inadequate, leading to the onset of glucose intolerance during pregnancy, a condition referred to as GDM [7].

Trimethylamine N-oxide (TMAO), dietary nutrients such as betaine, choline, and L-carnitine, and other choline-containing compounds are metabolized by the gut microbiota and various enzymes into trimethylamine (TMA) [8]. TMA is absorbed into the portal circulation via passive diffusion across enterocyte membranes and transported to the liver, where it is oxidized by hepatic flavin-containing monooxygenase 3 (FMO3) into trimethylamine N-oxide (TMAO) [9]. Approximately half of the generated TMAO is absorbed and subsequently excreted unchanged in the urine. The remaining fraction may be further metabolized into dimethylamine, formaldehyde, ammonia, and methane [10]. Emerging evidence also implicates gut microbiota-derived TMAO in the pathophysiology of various diseases, including cardiovascular disorders, preeclampsia, and diabetes mellitus [11,12].

Indoxyl sulfate (IS) is a uremic toxin formed through the bacterial metabolism of tryptophan in the gut. Specifically, the bacterial enzyme tryptophanase converts tryptophan into indole, which is subsequently absorbed into the bloodstream and transported to the liver, where it is further metabolized into indoxyl sulfate and excreted via urine [13].

Circulating levels of IS are significantly elevated in individuals with advanced chronic kidney disease (CKD) compared to those without renal impairment [14]. In diabetic patients, indoxyl sulfate seems to be a significant factor in determining redox balance. A strong relationship between the level of indoxyl sulfate in the blood and renal function, indicating that indoxyl sulfate elimination may be necessary starting in the early stages of diabetic nephropathy [15,16,17]. Given that diabetes profoundly alters the gut microenvironment and promotes a distinct microbial profile, it is plausible that diabetes may also be associated with elevated IS levels [18].

High-Sensitivity C-Reactive-Protein (Hs-CRP) is a non-immunoglobulin, pentameric acute-phase protein composed of five identical subunits. It is primarily synthesized by hepatocytes, though smaller amounts may also be produced by vascular smooth muscle cells and macrophages. It is commonly acknowledged that CRP is one of the most significant indicators of systemic inflammation [19]. It contributes to endothelial dysfunction by disrupting the vascular endothelial glycocalyx [20]. Furthermore, CRP promotes insulin resistance by serine phosphorylating the insulin receptor, which prevents phosphatidylinositol 3-kinase (PI3K) from communicating downstream of the receptor [21,22].

Numerous inflammatory indicators are strongly correlated with CRP levels such as interleukin-6 (IL-6) and fibrinogen and are elevated in individuals with impaired glucose tolerance as well as overt diabetes mellitus [23,24].

The development of the high-sensitivity CRP (Hs-CRP) assay has provided a valuable tool for detecting low-grade inflammation at the vascular level [25]. This assay employs ultrasensitive detection technology, allowing for accurate quantification of CRP at low concentrations and improving both the sensitivity and specificity of the measurement [26].

Maresin-1 (MaR-1), Maresin-1 (MaR-1) is biosynthesized in macrophages through the 14-lipoxygenation of docosahexaenoic acid (DHA) followed by enzymatic conversion of 13S,14S-epoxy intermediates [27,28]. Specialized pro-resolving mediators (SPMs) are a new family of bioactive lipid mediators that are produced from omega-3 polyunsaturated fatty acids, specifically eicosapentaenoic acid (EPA) and DHA [29]. Efferocytosis, the mechanism by which macrophages take up and eliminate apoptotic cells, is greatly aided by MaR-1 [30]. In addition, MaR-1 accelerates tissue regeneration, anti-inflammatory actions and repair processes. Clinical studies have demonstrated that MaR-1 exhibits promising therapeutic effects in the management of upper respiratory tract infections, pneumonia, and colitis, primarily by enhancing wound healing and mitigating diabetes-related complications [31]. Moreover, MaR-1 has been shown to reduce hyperalgesia and provide neuroprotective effects [32].

S-Equol (Testican-3) was first isolated in 1932 from horse urine [33]. It has since been identified in the urine and plasma of various animal species, including humans [34]. Equol exists in two enantiomeric forms: S-Equol and R-Equol [35]. Soy isoflavones are typically present in glycosylated form, and within the human and animal gut, intestinal microbiota convert the isoflavone daidzein into S-Equol through microbial fermentation [36]. Notably, only the S-enantiomer is produced in both humans and animals following the consumption of soy-based legumes [37]. Growing evidence suggests that S-Equol production may be associated with a decreased risk of metabolic disorders like obesity, insulin resistance, vascular dysfunction, hypertension, and type 2 diabetes.

Numerous studies have shown that diabetes is a chronic inflammatory illness that is significantly influenced by systemic inflammation [38,39,40]. Accordingly, the present study aimed to elucidate the potential associations between gestational diabetes mellitus (GDM) and key inflammatory and microbial metabolites, specifically TMAO, IS, Hs-CRP, MaR-1, and S-Equol.

## 2. Materials and Methods

This research was carried out as a collaborative medical specialty thesis under the supervision of Dr. Şeyda Yavuzkır from the Department of Obstetrics and Gynecology and Dr. Süleyman Aydın from the Department of Medical Biochemistry at Fırat University, Elazig, Turkiye. Ethical approval was obtained from the Ethics Committee of Fırat University Rectorate, Elazig, Turkiye (Decision No: 2023/14–29, dated 14 December 2023). In accordance with the principles of the Declaration of Helsinki, written informed consent was obtained from all participants prior to the collection of blood samples for biochemical analyses.

Sixty pregnant women were gathered from the Department of Obstetrics and Gynecology at Fırat University Medical Faculty Hospital, including 30 patients diagnosed with gestational diabetes mellitus (GDM) and 30 healthy pregnant controls.

The International Federation of Gynecology and Obstetrics, the World Health Organization (WHO), the Endocrine Society, and the American Diabetes Association (ADA) now recommend using the International Association of Diabetes and Pregnancy Study Group (IADPSG) criteria to diagnose GDM [41]. The diagnosis of diabetes was made based on the diagnostic criteria for diabetes and prediabetes established by the IADPSG [41,42]. Since a value of 92 mg/dL or higher is indicative of GDM, the IADPSG advises that all women have a fasting plasma glucose (FPG) test at their first prenatal appointment. Women whose FPG is less than 92 mg/dL should have a 2 h 75 g oral glucose tolerance test (OGTT) between 24 and 28 weeks of gestation. The pregnant women were divided into two groups for evaluation:

Group 1 (Control group): Pregnant women without a prior or current diagnosis of diabetes, free of chronic or systemic illnesses, who had not experienced any pregnancy-related complications and who tested negative for GDM via 75 g oral glucose tolerance test (OGTT) performed between gestational weeks 24 and 28.

Group 2 (GDM group): Pregnant women without a history of chronic illness who were diagnosed with GDM using the 75 g OGTT between weeks 24 and 28, and who delivered vaginally at term without complications.

The presented study is a case–control as the participants are initially grouped based on the outcome of interest which is GDM. The diagnostic criteria set forth by the IADPSG for diabetes and prediabetes were used as the primary reference standard [41]. Pregnant women with pre-existing family and personal history of GDM, chronic hypertension, thromboembolic disease, thrombophilia, insulin resistance, such as polycystic ovarian syndrome (PCOS), women of advanced maternal age, those who are overweight/obese, use of saturated fats and refined sugars, hepatic or renal disorders, or known fetal anomalies, alcohol use intense physical activity were excluded from the study. Also, participants who required antibiotic therapy and supplement use during pregnancy were not included. Women included in the control group (Group 1) had no history of diabetes in their current or previous pregnancies and had no significant obstetric complications (e.g., placenta previa, intrauterine growth restriction, placental abruption, comorbidities (HTN, renal/hepatic disease) in their obstetric history.

Detailed obstetric histories were obtained from all participants, and comprehensive obstetric evaluations were performed. All pregnant women were placed on a standardized dietary regimen, with macronutrient distribution maintained at 55–60% carbohydrates, 25–30% fats, and 12–15% proteins [43]. Data collected included maternal age, gestational week, blood pressure measurements, and previous pregnancy history, if applicable.

In the GDM group, blood glucose levels were initially managed through lifestyle modifications and light physical activity. Pharmacologic therapy (insulin or, in rare cases, metformin) was initiated only if glycemic control could not be achieved through non-pharmacological interventions. However, participants who required pharmacologic treatment with insulin or metformin were excluded from the final analysis.

### 2.1. Collection and Storage of Biological Samples

Following the diagnosis of GDM and classification of healthy controls, venous blood samples (10 mL) were collected from all pregnant participants in both the control and GDM groups using plain biochemistry tubes at two time points: prepartum and 4 h postpartum as previously described [44]. The serum was extracted from the samples by centrifuging them for five minutes at 4000 rpm (1792 g-force). It was then aliquoted and kept at −80 °C until biochemical assays were completed.

### 2.2. The Manufacturer Information and Analysis of Biological Samples via ELISA

The characteristics of the ELISA kits used in the study and the manufacturer information are briefly as follows.

- **TMAO:** Human TMAO ELISA Kit (Shanghai Sunredbio Technology Co., Ltd., Shanghai, China, Cat. No: 201-12-7378); sensitivity: 0.043 ng/mL, detection range: 0.05–10 ng/mL
- **Indoxyl Sulfate (IS):** Human IS ELISA Kit (Shanghai Sunredbio Technology Co., Ltd., Shanghai, China, Cat. No: 201-12-7596); sensitivity: 1.854 µg/mL, detection range: 2–600 µg/mL
- **Maresin-1 (MaR-1):** Human MaR-1 ELISA Kit (Shanghai Sunredbio Technology Co., Ltd., Shanghai, China, Cat. No: 201-12-7339); sensitivity: 28.625 ng/L, detection range: 30–9000 ng/L
- **S-Equol:** Human S-Equol ELISA Kit (Shanghai Sunredbio Technology Co., Ltd., Shanghai, China, Cat. No: 201-12-8142); sensitivity: 0.247 ng/mL, detection range: 0.25–70 ng/mL
- **High-Sensitivity CRP (hs-CRP):** Human hs-CRP ELISA Kit (Shanghai Sunredbio Technology Co., Ltd., Shanghai, China, Cat. No: 201-12-1806); sensitivity: 0.112 mg/L, detection range: 0.15–40 mg/L

The intra-assay coefficients of variation (CV) for all ELISA kits used were below 10%, and inter-assay CVs were below 15%, indicating acceptable analytical reproducibility and precision.

The serum levels of TMAO, IS, MaR-1, S-Equol and hs-CRP were made by using enzyme-linked immunosorbent assay (ELISA) kits in accordance with the manufacturer’s instructions and Aydin and his-coworkers’ instructions [44]. Briefly, the ELISA procedure is as follows: A particular antibody that is unique to the molecules to be analyzed is applied to the microelisa plate that comes with the kit. The particular antibody is mixed with samples that have been sown into the wells. After that, the wells are filled with a particular Horseradish Peroxidase (HRP)-conjugated antibody for the quantification of the molecules, and they are incubated. Following incubation, the Bio-Tek ELX50 automatic washer (BioTek Instruments, Winooski, VT, USA) was used to empty each well and wash it five times with wash buffer. Chromogenic substrates are applied to each well following washing. When the stop solution is added, the blue color will change to yellow [44]. Then, optical density measurements (at 450 nm wave) were conducted using a ChroMate microplate reader (Awareness Technology Inc., Palm City, FL, USA).

### 2.3. Statistical Analysis

SPSS (Statistical Package for the Social Sciences) version 22 (SPSS Inc., Chicago, IL, USA) was used for all statistical analyses. For categorical variables, descriptive statistics were displayed as frequencies and percentages; for continuous variables, they were displayed as mean ± standard deviation (mean ± SD). The Kolmogorov–Smirnov test was used to determine whether continuous variables had a normal distribution. When comparing two groups, normally distributed variables were compared using the independent samples *t*-test, while non-normally distributed variables were compared using the Mann–Whitney U test (nonparametric). For normally distributed data, the paired samples *t*-test was utilized to compare molecular parameters before and after delivery (prepartum vs. postpartum); in other cases, the Wilcoxon signed-rank test was employed. To assess correlations between continuous variables, Spearman’s rank correlation coefficient was used. Statistical significance was defined as a *p*-value of less than 0.05.

## 3. Results

The study initially enrolled 60 participants. However, due to participant withdrawals (Some participants objected to the usage of their data after the study was over. Participants have a right to leave the research at any moment and without explanation in accordance with our IRB regulations), and exclusion criteria, the final analysis was conducted on 44 individuals: 22 women diagnosed with GDM and 22 healthy pregnant controls. Pregnant women who left the study had no impact on the findings, per the statistical analysis of G-power. The GDM group’s mean age was 33.1 ± 5.2 years, while the control group’s was 30.7 ± 4.6 years. There was no statistically significant difference between the two groups (*p* = 0.113). Fetal weight at the time of OGTT (*p* < 0.001) and birth week at delivery (*p* = 0.005) were significantly lower in the GDM group compared to controls. Also, birth weight in the GDM group was significantly lower than in the control group (*p* = 0.001) (Table 1).

**Figure 1:**
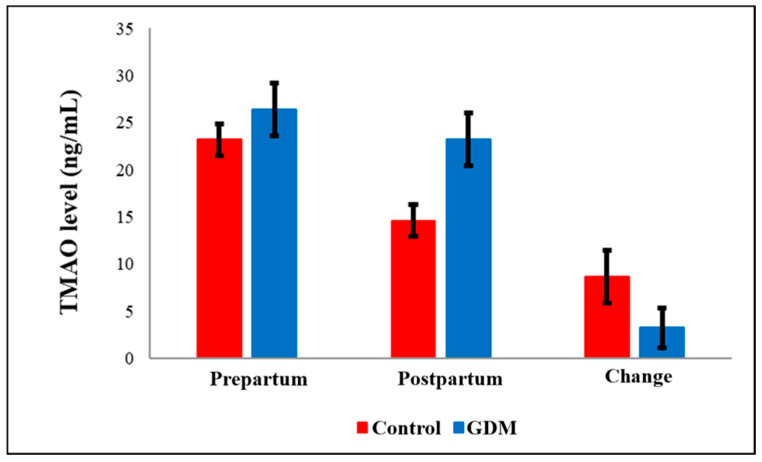
Comparison of TMAO levels by groups. GDM = Gestational Diabetes Mellitus. TMAO = Tri-metilamin-N-Oksit.

**Figure 2:**
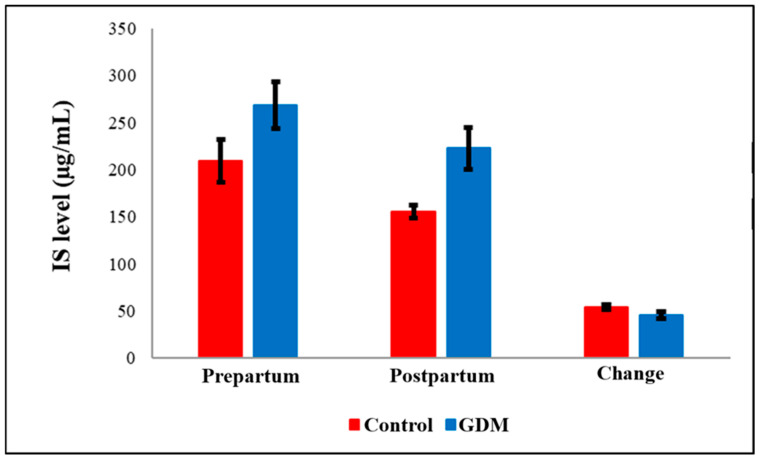
Comparison of IS levels by groups. GDM = Gestational Diabetes Mellitus. IS = Indoxyl Sulfate.

**Figure 3:**
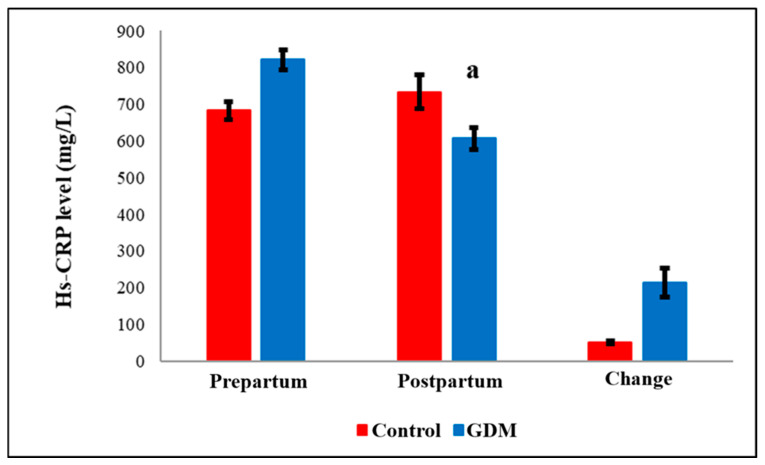
Comparison of CRP levels by groups. CRP = C-Reactive Protein. GDM = Gestational Diabetes Mellitus. a: GDM Prepartum group versus GDM Postpartum group (*p* = 0.019).

TAMO (Figure 1) and IS (Figure 2) metabolic markers are lower in post-partum compared to pre-partum but not significantly changed within GDM group. However, testing these within group comparison by paired *t*-test showed significant drop within controls but not within GDM. Also, Hs-CRP (Figure 3) were elevated in the GDM group compared to controls, these differences did not reach statistical significance. However, within the GDM group, postpartum Hs-CRP levels were significantly lower than their prepartum levels (*p* < 0.05, Figure 3).

**Figure 4:**
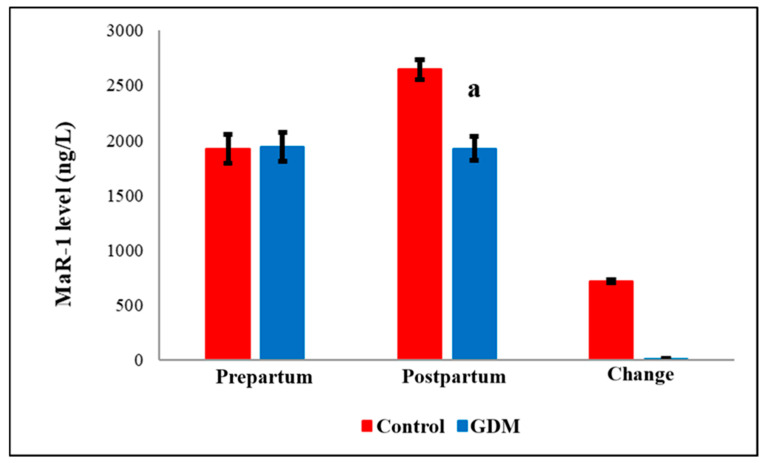
Comparison of MaR-1 levels by groups. GDM = Gestational Diabetes Mellitus. MaR-1 = Maresin-1. a: GDM Postpartum group versus Control Postpartum group (*p* = 0.036).

**Figure 5:**
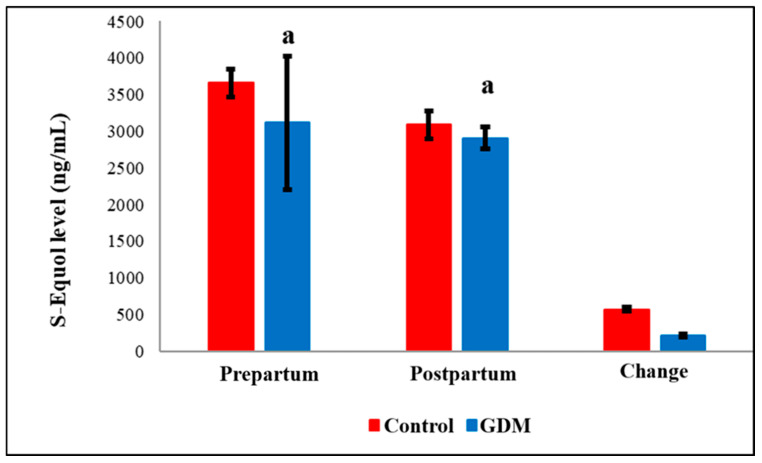
Comparison of S-Equol levels by groups. GDM = Gestational Diabetes Mellitus. a: GDM Prepartum group versus Control Prepartum group (*p* = 0.041). a: GDM Postpartum group versus Control Postpartum group (*p* = 0.048).

Notably, serum post-partum levels of Maresin-1 (MaR-1, Figure 4) and S-Equol (Figure 5) were significantly reduced in the GDM group compared to healthy controls (*p* = 0.036 and *p* < 0.05, respectively). The detailed comparisons of prepartum and postpartum values, along with their corresponding changes, are illustrated in Figure 1, Figure 2, Figure 3, Figure 4 and Figure 5. Additionally, within the GDM group, there was a statistically significant positive connection between prepartum S-Equol levels and prepartum MaR-1 (Table 2).

## 4. Discussion

GDM is a complex condition associated with increased neonatal morbidity, shoulder dystocia, premature birth, polyhydramnios, and fetal abnormalities [45,46]. In this investigation, it was shown for the first time that serum levels of Maresin-1 (MaR-1) and S-Equol were significantly decreased in GDM patients, whereas levels of TMAO, indoxyl sulfate (IS), and hs-CRP were partially elevated but not statistically significant.

Previous studies have reported that serum TMAO levels are significantly elevated in individuals with diabetes compared to healthy controls [47]. In another study, a 372 ng/mL increase in plasma TMAO concentration was associated with a 54% rise in the prevalence of diabetes mellitus [48]. Although the GDM group’s prepartum and postpartum TMAO levels were higher than those of the controls in our investigation, the differences were not statistically significant. Measurement of metabolites of TMAO such as ammonia in serum and urine post-partum and shortly after to ensure their levels went down to normal range [49]. It seems that these sustained levels after delivery are associated with GDM.

Furthermore, it is commonly known that dietary consumption of choline and other TMAO precursors, and carnitine significantly affects circulating and urinary TMAO levels [50]. To control for this, macronutrient composition in all participants’ diets was standardized, with 55–60% of energy from carbohydrates, 25–30% from fats, and 12–15% from proteins. The absence of a statistically significant increase in TMAO levels may be attributable to this controlled dietary regimen, which likely reduced the abundance or activity of TMA-producing gut bacteria.

Moreover, alterations in gut microbiota composition during late pregnancy and postpartum may influence TMAO biosynthesis. In animal models, the presence of specific gut microbes has been shown to facilitate TMA production, which is then oxidized to TMAO in the liver by flavin-containing monooxygenase 3 (FMO3) [51]. A shift in microbial populations due to the controlled diet could have suppressed TMA-producing strains, thereby lowering TMAO biosynthesis. Additionally, the exact relationship between TMAO and diabetes remains controversial. However, TMAO has been implicated in promoting insulin resistance by binding and activating the PERK protein [52]. Additionally, there is evidence that dietary TMAO may contribute to the pathophysiology of type 2 diabetes by causing inflammation in adipose tissue and disrupting hepatic insulin signaling [53].

While our study also found elevated IS levels in GDM patients compared to controls, a statistically significant difference did not exist. Under pathological conditions characterized by increased lipid and ketone body metabolism, such as diabetes, high-fat diets, or fasting, the expression of cytochrome P450 2E1 (CYP2E1) increases, contributing to the hepatic transformation of indole (a tryptophan metabolite) into IS [54]. Wakabayashi et al. reported elevated urinary IS levels in diabetic patients, likely reflecting oxidative stress [55]. Similarly, Patney et al. observed increased urinary IS in diabetic patients and in those with diabetic neuropathy, particularly in individuals presenting with steatorrhea [56]. All of these results point to IS as a potential new indicator of oxidative stress in GDM and other metabolic diseases.

In our study, although pre-partum Hs-CRP amount were higher in GDM patients compared to controls, the increase was not statistically significant. Prior research has generally shown that CRP levels rise during pregnancy and are further elevated in GDM [57]. Elevated CRP has also been reported in patients with T2DM, independent of BMI and insulin resistance [24]. The liver produces CRP, an acute-phase reactant, in reaction to inflammatory stimuli, and its serum concentration can increase up to 1000-fold during infection or tissue injury [58].

One possible explanation for the lack of a significant CRP increase in our GDM cohort is the incomplete depletion of endogenous anti-inflammatory lipid mediators such as MaR-1. In our study, MaR-1 levels were reduced but not fully depleted in GDM patients, suggesting that residual MaR-1 may still exert a suppressive effect on CRP synthesis. The anti-inflammatory role of MaR-1 is supported by previous studies showing that IL-1β inhibition improves glycemic control in animal models of GDM [59]. Therefore, we propose that molecules like MaR-1 may partially modulate the inflammatory cascade, thereby preventing a marked rise in CRP levels. Nevertheless, validation of this hypothesis will require corroboration by independent laboratories.

Importantly, MaR-1 levels were significantly lower in the GDM group compared to controls in our study. MaR-1 is an endogenous lipid mediator derived from polyunsaturated fatty acids and exhibits both antioxidant and anti-inflammatory properties. Chronic inflammation is known to have a part in the etiology of diabetes and its consequences. Anti-inflammatory mediators like MaR-1 have been shown to mitigate disease progression by modulating insulin resistance and adipokine secretion [60]. In high-fat diet-induced obese mice, MaR-1 treatment significantly reduced proinflammatory cytokines such as TNF-α and IL-1β [61]. Moreover, in experimental models of diet-induced hyperglycemia, MaR-1 administration restored glucose levels to near normal [62]. MaR-1 has also been shown to enhance macrophage-mediated tissue repair and promote diabetic wound healing [63]. These findings suggest that MaR-1 may serve as a promising diagnostic and therapeutic biomarker for GDM. Restoration of MaR-1 levels to physiological ranges may offer a novel strategy to prevent GDM-associated complications. Further clinical trials are needed to evaluate its therapeutic applicability.

Similarly, our research showed that GDM patients had considerably lower S-Equol levels than controls. Prior research has shown that S-Equol prevents oxidative stress-induced apoptosis in pancreatic beta cells. It is yet unknown, however, if S-Equol improves insulin secretion, beta-cell proliferation, or general function in vivo [64]. Another study found that giving mice oral S-Equol dramatically reduced the frequency of streptozotocin-induced hyperglycemia [65]. Usui et al. reported that S-Equol supplementation (10 mg/day) reduced glycated hemoglobin concentrations in S-Equol non-producers [66]. By maintaining β-cell mass and insulin secretion, S-Equol has also been demonstrated to prevent streptozotocin-induced hyperglycemia. This is most likely due to a combination of increased cell proliferation and inhibited apoptosis [67]. Collectively, these findings suggest that S-Equol enhances both insulin secretion and β-cell preservation, indicating its therapeutic potential in diabetes management. Therefore, the significant reduction in S-Equol in GDM patients highlights the possibility that restoring its physiological levels may contribute to alleviating GDM-related complications.

There are various limitations to this study. Most notably, some participant removal resulted in a smaller sample size, which may have impacted statistical power. Another limitation of this study is that the microbiota composition was not examined microbiologically. The necessity to account for women from diverse ethnic, socioeconomic, and dietary backgrounds is another limitation of our research for broader generalizing. Additionally, the study was limited to pregnant women; thus, future studies should include both male and female participants and examine similar parameters in cases of type 1 diabetes, type 2 diabetes, and idiopathic diabetes for broader applicability.

## 5. Conclusions

The current study concluded by showing that there may be connections between GDM and particular inflammatory biomarkers (Maresin-1, high-sensitivity C-reactive protein), as well as microbiota-derived metabolites (Trimethylamine-N-oxide, S-Equol, and Indoxyl Sulfate), in patients with gestational diabetes mellitus. In addition to the aforementioned molecules, GDM is linked to a higher concentration of resident adipose tissue macrophages (ATM), which release proinflammatory cytokines such as TNF-α, IL-6, and IL-1β. These findings imply that although inflammation seems to have a significant role in the etiology of GDM, the connection between these molecules may not be clear-cut. Therefore, leptin, ghrelin, irisin, and so on, as well as cytokines, should be studied simultaneously in the future to better understand the pathophysiology of GDM. In addition to these restrictions, these findings suggest that MaR-1 and S-Equol may be helpful biochemical biomarkers for GDM tracking and early identification.

## Figures and Tables

**Table 1:** Comparison of age and pregnancy characteristics by groups.

Parameters	Control (*n* = 22)	GDM (*n* = 22)	*p*
Age (year)	30.7 ± 4.6	33.1 ± 5.2	0.113 *
Fetus weight (g)	1285.2 ± 121.9	1052.5 ± 210.3	<0.001 *
Birth week	37.5 ± 1.2	35.2 ± 2.8	0.005 **
Birth weight (g)	3555.7 ± 320.3	2945.0 ± 667.3	0.001 **
Pre-pregnancy BMI (kg/m^2^)	23.8 ± 2.7	24.3 ± 2.9	0.372 *
Maternal BMI (kg/m^2^)	30.0 ± 3.5	31.0 ± 3.1	0.306 *
Weigh gain during pregnancy (kg)	12.9 ± 1.1	13.7 ± 1.7	0.47
Parity	2	2	……
Family history of diabetes	None	None	……

* Student *t* test, ** Mann–Whitney U analysis was applied. BMI = Body Mass Index. GDM = Gestational Diabetes Mellitus.

**Table 2:** Correlation analysis in the participant (GDM) group (*n* = 22).

Parameters		Age	Fetus Weight	Birth Week	Birth Weight	Maternal BMI	Pre TMAO	Pre IS	Pre Hs-CRP	Pre MaR-1
Fetus weight	r	−0.037								
	*p*	0.871								
Birth week	r	0.163	−0.417							
	*p*	0.470	0.054							
Birth weight	r	0.262	0.395	−0.230						
	*p*	0.240	0.069	0.304						
Maternal BMI	r	−0.128	−0.123	−0.282	−0.035					
	*p*	0.572	0.586	0.203	0.877					
Pre TMAO	r	−0.140	0.064	0.111	−0.121	−0.019				
	*p*	0.534	0.776	0.624	0.592	0.934				
Pre IS	r	−0.015	0.118	−0.339	−0.028	0.340	0.256			
	*p*	0.946	0.602	0.123	0.902	0.122	0.249			
Pre Hs-CRP	r	−0.037	−0.368	0.191	0.019	0.081	0.049	−0.250		
	*p*	0.869	0.092	0.395	0.934	0.721	0.828	0.262		
Pre MaR-1	r	−0.003	−0.031	0.158	−0.133	−0.163	0.201	0.188	−0.003	
	*p*	0.988	0.893	0.483	0.556	0.469	0.371	0.402	0.990	
Pre S-Equol	r	0.119	0.034	0.021	−0.075	−0.086	0.154	0.239	0.025	0.874
	*p*	0.598	0.880	0.925	0.741	0.702	0.493	0.283	0.911	0.000

BMI = Body Mass Index, Hs-CRP = High-Sensitivity C-Reaktif Protein. IS = Indoxyl Sulfate. MaR-1 = Maresin-1. Pre: Prepartum. TMAO = Tri-metilamin-N-Oksit.

## Data Availability

The data supporting the findings of this study are available from the corresponding author upon reasonable request. Due to ethical restrictions and concerns regarding patient confidentiality, the data are not publicly accessible.

## Abbreviations

The following abbreviations are used in this manuscript:

Abbreviation	Full Term
CRP	C-reactive protein
DHA	Docosahexaenoic acid
DM	Diabetes mellitus
EPA	Eicosapentaenoic acid
FUBAP	Firat university scientific research projects unit
GDM	Gestational diabetes mellitus
Hs-CRP	High-sensitivity c-reactive protein
IS	Indoxyl sulfate
MaR-1	Maresin-1
RAAS	Renin–angiotensin–aldosterone system
SPM	Specialized pro-resolving mediators
TMA	Trimethylamine
TMAO	Trimethylamine n-oxide
Th1	T-helper 1 cell
